# Terpenes

**DOI:** 10.3762/bjoc.15.292

**Published:** 2019-12-13

**Authors:** Jeroen S Dickschat

**Affiliations:** 1 Kekulé-Institut für Organische Chemie und Biochemie, Rheinische Friedrich-Wilhelms-Universität Bonn, Gerhard-Domagk-Straße 1, D-53121 Bonn, Germany

**Keywords:** Terpenes

With more than 80,000 known compounds terpenes represent the largest class of natural products. Their remarkable structural complexity and diversity is a result of a unique biosynthetic pathway invented by nature that starts with the formation of only a few acyclic precursors termed oligoprenyl diphosphates. These precursors containing multiple olefinic double bonds can be ionised to generate a highly reactive cationic intermediate that can be subject to a cascade reaction through typical carbocation chemistry, including cyclisation reactions, hydride migrations and Wagner–Meerwein rearrangements [1–2]. The cascade is usually terminated by deprotonation or attack of water. The initially formed terpene hydrocarbons can subsequently be modified by enzymatic oxidations that often involve radical chemistry.

The non-functionalised hydrocarbons are volatile and often exhibit interesting odour properties [3]. As a consequence, these compounds may act as chemical signals such as pheromones, but also have a long standing history as fragrances used by humans. The oxidised derivatives may be of high interest because of their bioactivity, with some of the most important drugs used in medical applications being of terpenoid origin. Two of the most fanous examples include the diterpenoid taxol from *Taxus brevifolia* that is used to treat breast and other types of solid cancers, while artemisinin from *Artemisia annua* is used as a cure against malaria [4].

This thematic issue covers all different aspects of terpene chemistry and biochemistry, including compound isolation and structure elucidation, total synthesis, biosynthetic studies, bioactivity and functionalisation for mode of action studies, and other applications of terpenes, e.g. as fragrances. I thank all colleagues who have participated in this issue for their contributions and the whole Beilstein team for the professional support, and I wish the readers of the issue some joyful and stimulating new insights.

Jeroen S. Dickschat

Bonn, December 2019
